# Tryptophan-kynurenine pathway attenuates β-catenin-dependent pro-parasitic role of STING-TICAM2-IRF3-IDO1 signalosome in *Toxoplasma gondii* infection

**DOI:** 10.1038/s41419-019-1420-9

**Published:** 2019-02-15

**Authors:** Tanmay Majumdar, Shagun Sharma, Manmohan Kumar, Md. Arafat Hussain, Namita Chauhan, Inderjeet Kalia, Amit Kumar Sahu, Vipin Singh Rana, Ruchi Bharti, Arun Kumar Haldar, Agam P. Singh, Shibnath Mazumder

**Affiliations:** 1 Immunobiology Laboratory, Department of Zoology, University of Delhi, Delhi, 110007 India; 2 Department of Zoology, University of Delhi, Delhi, 110007 India; 3 Infectious Diseases Laboratory, National Institute of Immunology, Aruna Asaf Ali Marg, Delhi, 110067 India; 4 Division of Biochemistry, CSIR-Central Drug Research Institute, Sector-10, Jankipuram Ext., Lucknow, 226031 India

## Abstract

Recent studies have documented the diverse role of host immunity in infection by the protozoan parasite, *Toxoplasma gondii*. However, the contribution of the β-catenin pathway in this process has not been explored. Here, we show that AKT-mediated phosphorylated β-catenin supports *T. gondii* multiplication which is arrested in the deficiency of its phosphorylation domain at S552 position. The β-catenin-TCF4 protein complex binds to the promoter region of IRF3 gene and initiates its transcription, which was also abrogated in β-catenin knockout cells. TBK-independent phosphorylation of STING(S366) and its adaptor molecule TICAM2 by phospho-AKT(T308S473) augmented downstream IRF3-dependent IDO1 transcription, which was also dependent on β-catenin. But, proteasomal degradation of IDO1 by its tyrosine phosphorylation (at Y115 and Y253) favoured parasite replication. In absence of IDO1, tryptophan was catabolized into melatonin, which supressed cellular reactive oxygen species (ROS) and boosted parasite growth. Conversely, when tyrosine phosphorylation was abolished by phosphosite mutations, IDO1 escaped its ubiquitin-mediated proteasomal degradation system (UPS) and the stable IDO1 prevented parasite replication by kynurenine synthesis. We propose that *T. gondii* selectively utilizes tryptophan to produce the antioxidant, melatonin, thus prolonging the survival of infected cells through functional AKT and β-catenin activity for better parasite replication. Stable IDO1 in the presence of IFN-γ catabolized tryptophan into kynurenine, promoting cell death by suppressing phospho-AKT and phospho-β-catenin levels, and circumvented parasite replication. Treatment of infected cells with kynurenine or its analogue, teriflunomide suppressed kinase activity of AKT, and phosphorylation of β-catenin triggering caspase-3 dependent apoptosis of infected cells to inhibit parasite growth. Our results demonstrate that β-catenin regulate phosphorylated STING-TICAM2-IRF3-IDO1 signalosome for a cell-intrinsic pro-parasitic role. We propose that the downstream IRF3-IDO1-reliant tryptophan catabolites and their analogues can act as effective immunotherapeutic molecules to control *T. gondii* replication by impairing the AKT and β-catenin axis.

## Introduction

*Toxoplasma gondii*, an intracellular coccidium that has been coevolving with vertebrates over billions of years, commonly infects warm-blooded animals including humans^1^. In humans, *T. gondii* is acquired by ingestion of either tissue cysts in infected meat or oocysts in food contaminated with cat faeces.

*T. gondii* modulates a number of cell survival pathways to promote its replication and infection in host cells. In canonical Wnt-mediated signalling which is one of the major survival pathways, the serine-threonine protein kinase, AKT, phosphorylates β-catenin at Ser552 phosphosite^2–4^, as a result, cytosolic phospho-β-catenin accumulates and enters the nucleus to interact with T cell factor/lymphoid enhancer-binding factor (TCF/LEF) family of transcription factors to promote transcription of several target genes^5–7^. Accumulating evidence has suggested that crosstalk between *T. gondii* infection and Wnt/β-catenin pathway regulates host gene expression^8,9^. However, the exact role of this pathway in controlling cellular innate immune response remained unexplored.

We previously observed, *T. gondii* infection activated intracellular nucleic acid sensor, STING, and STING-TRIF heterodimer activated downstream TANK-binding kinase 1 (TBK1) to phosphorylate IRF3 for enhancing parasitic growth in host ^10,11^. Phosphorylation of both STING and TRIF was indispensable for IRF3 induction^12^. TIR containing adaptor molecule-2 (TICAM2) is an alternative adaptor molecule, involved in IRF3 activation. Previous studies have shown that β-catenin-IRF3 complex binds to the promoter region of IFN-β^13,14^. However, under certain conditions, IRF3 independent IFN expression occurred through TCF binding sites present at the IFN-promoter^15^. Here, we show that the DNA-binding sites of phospho-β-catenin-TCF4 are present in the human IRF3 promoter region and β-catenin phosphorylation at S552 induces IRF3 transcription. Phospho-IRF3 is known to induce several interferon stimulated genes (ISGs), including indoleamine-pyrrole-2,3-dioxygenase-1/2 (IDO1/2)^16^. Tryptophan can be catabolised either by tryptophan 2,3-dioxygenase (TDO), IDO1 or IDO2^17–20^. While IDO2 is mostly expressed in kidney, and TDO in liver^21^, IDO1, upregulated by interferon gamma (IFN-γ), is the predominant enzyme found in a variety of cells, including epithelial cells, macrophages, microglia, neurons and astrocytes^22–26^. Several earlier studies have suggested that IDO1 activation by IFN-γ impedes *T. gondii* growth^27–29^. Interestingly, in absence of IDO1/2 or TDO, tryptophan is catabolized to melatonin by a parallel pathway. A well-known scavenger of ROS, melatonin promotes cell survival by increased AKT activity^30^. Natural infection by *T. gondii* occurs through oral ingestion, leading to infection of intestinal epithelial cells^31^. In this study, we have, therefore, used human colon adenocarcinoma cell line Caco2 to decipher the mechanism of *T. gondii* infection. Caco2 cells develop apical polarity and junctional complexes, characteristic of human enterocytes, thereby serving as suitable host cells to explore the mechanism of *T. gondii* infection^32,33^. Here, we report that *T. gondii* infection in Caco2 cells leads to phosphorylation of several molecules such as β-catenin, STING, and its adaptor molecule TICAM2 by AKT. STING-TICAM2 heterodimer activates downstream phospho-IRF3 mediated IDO1 transcription, leading to an intricate signalling network that connects tryptophan catabolism and apoptosis to impede parasite replication.

## Results

### Phosphorylation of β-catenin facilitates *T. gondii* replication

**Fig. 1:**
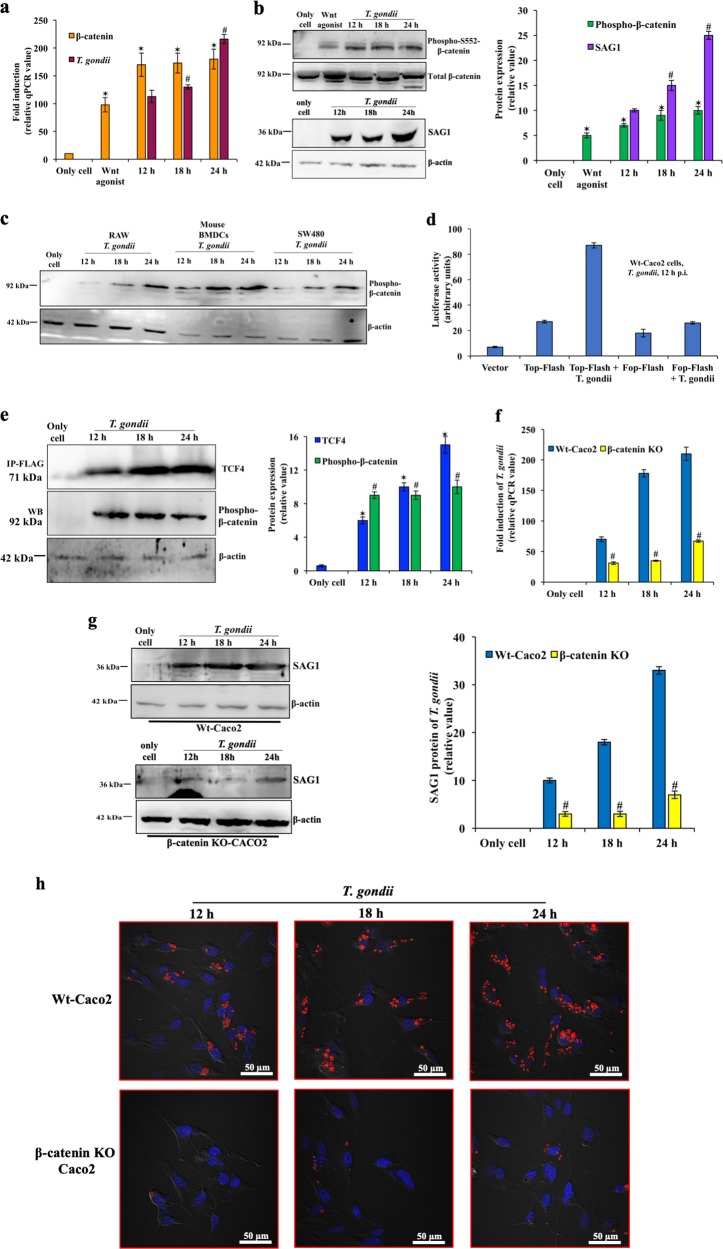
Phosphorylation of β-catenin helps *T. gondii* replication. **a** Human colon cell line, Caco2 was infected with *T. gondii*, and parasite growth at indicated time post-infection was quantified by qPCR of genomic DNA using the invariant ITS-1 primers, normalized against β-actin. Expression of β-catenin was also measured by qPCR. **b** Phospho-β-catenin (92 kDa) and *T. gondii* SAG1 (36 kDa) protein at the indicated time post-infection were analysed by immunoblotting. The intensity of each band was plotted in bar diagram after normalizing against total β-catenin (92 kDa) or β-actin (42 kDa). **c** Raw macrophage cell lines, mouse bone-marrow dendritic cells (BMDCs), and human colon epithelial cells (SW480) were separately infected with *T. gondii* (36 kDa) and phospho-β-catenin (92 kDa) was monitored by immunoblot. β-actin served as internal control. **d** Luciferase reporter assays of TCF/LEF was done after *T. gondii* infection. Caco2 cells were transfected with Vector (pGL3), or TOP-Flash, or FOP-Flash reporter plasmid with internal control pCMV-Renilla-Luc plasmid for 8 h. At 12 h post-infection, cells were harvested for dual luciferase assay. The firefly luciferase units were normalized against the Renilla units and expressed in percentage. **e** FLAG-TCF4 from transfected cells was immunoprecipitated with FLAG antibody after infection, and the Western blot (WB) was done with FLAG (TCF4 expression, 71 kDa), phospho-β-catenin (92 kDa), and β-actin antibody (42 kDa). In the right panel, the bar diagram represents the average expression levels of FLAG-TCF4, and phospho-β-catenin of immunoprecipitated samples of three biological repeats after normalizing with β-actin. **f** *T. gondii* growth was quantified by qPCR using the invariant ITS-1 primers in wild-type (Wt) and β-catenin knock-out (KO) cells. **g** Replication kinetics of *T. gondii* was measured in both Wt and β-catenin KO cells by immunoblot. The intensities of the SAG1 bands (36 kDa) are plotted in bar diagram (right panel). Only cells denote uninfected cells with 2 µl of vehicle (DMSO or lipofectamine), Wnt agonist = AMBMP hydrochloride (20 µM, DMSO). Mean values ± SE of three experiments are shown here. Values were averaged from three separate experiments for each diagram, with error bars shown. *p < 0.05 and ^#^*p* < 0.01. **h** Wt and β-catenin KO cells were infected by mCherry expressing *T. gondii* RH at an MOI of 2, and at the indicated times (12, 18, 24 h) stained for nucleus (DAPI, blue) and visualized by confocal microscopy. Scale bar = 50 µm

We found enhanced growth of *T. gondii* concomitant to higher expression of β-catenin **(***CTNNB1*) mRNA at indicated time of infection (Fig. 1a). To determine β-catenin function, we observed increasing β-catenin-Ser552(S552) phosphorylation along with total β-catenin was correlated with increasing *T. gondii* replication (Fig. 1b). Wnt agonist, AMBMP hydrochloride (20 µM), was used as a positive control. To test the universality of this phenomenon, diverse cells were used and similar pattern of increased phospho-β-catenin was observed (Fig. 1c). *T. gondii* infection also promoted transcription of TCF. Caco2 cells were transfected with Top-Flash, followed by 12 h post-infection, resulting in enhanced transcriptional activation of a reporter gene with multiple copies of upstream TCF-binding sites, whereas mutation of TCF/LEF binding sites (Fop-Flash) abrogated its transcriptional activation during infection (Fig. 1d). To test the involvement of TCF4 in β-catenin pathway, cells were transfected with FLAG-TCF4 plasmid, then immunoprecipitated using FLAG antibody after parasite infection. We found that FLAG-TCF4 levels increased in course of infection. Moreover, β-catenin, which complexed with TCF4, was found to be phosphorylated at S552 (Fig. 1e). These results documented that *T. gondii* infection upregulates both β-catenin and TCF, and we hypothesized that their heterodimeric complex is required for several downstream pathways.

To test whether presence of β-catenin is indispensable for parasite growth, we infected both wild-type (Wt) and β-catenin knockout (KO) cells and noted that parasite replication was abrogated in absence of β-catenin (Figs. 1f–h). In wild-type cells, intracellular parasites load was higher (Fig. 1h), and intracellular parasitophorous vacuoles (PVs) were distinct compared to β-catenin KO cells (Supplementary Fig. S1a).

### AKT is required for β-catenin phosphorylation during *T. gondii* growth

**Fig. 2:**
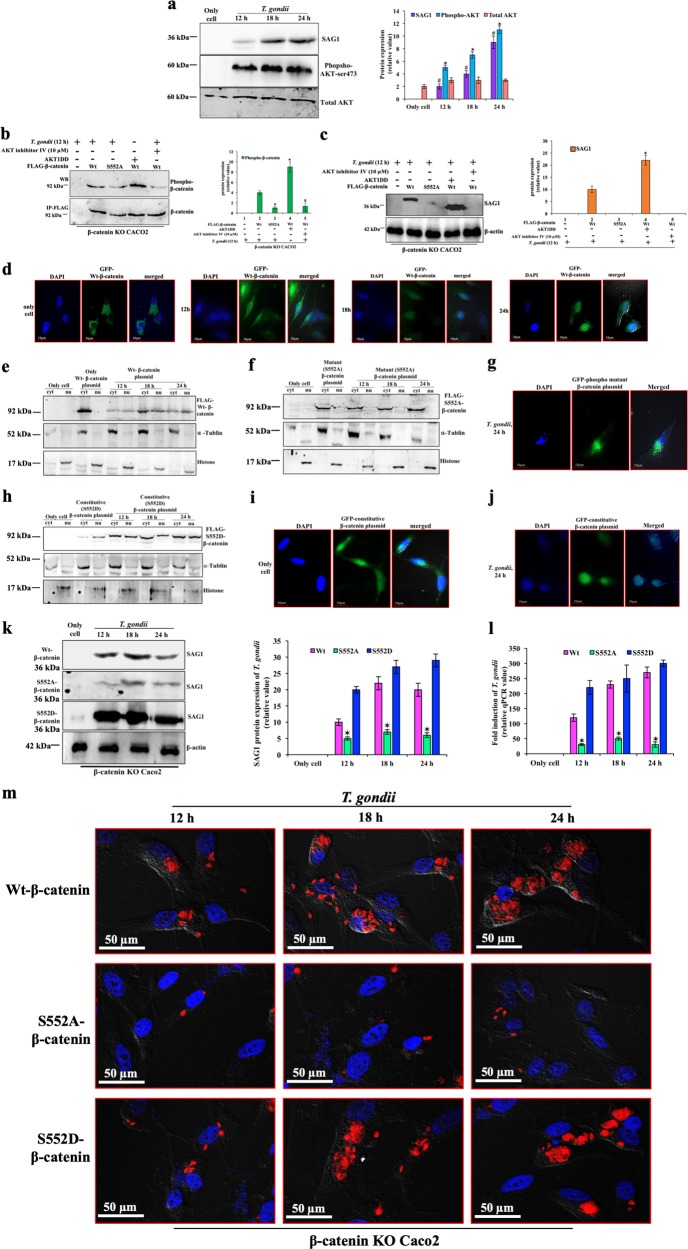
AKT dependent phosphorylation of β-catenin is indispensable for its nuclear translocation in *T. gondii* infection. **a** After infection, Caco2 cells were lysed and immunoblotting was done using *T. gondii* SAG1 (36 kDa) and phospho-AKT antibody (60 kDa). We used total AKT (60 kDa) as internal control and mean intensity of each band of three biological repeats of these blots were represented by bar diagram at the right panel. **b** β-catenin KO cells were transfected with FLAG-wild-type (Wt), FLAG-phospho-mutant (S552A) β-catenin plasmids in presence or absence of either AKT inhibitor, or co-transfected with constitutive phospho-mimic AKT1 (HA-AKT1DD) plasmid. All samples were infected with parasite for 12 h, except cells of Wt-β-catenin with HA-AKT1DD, and immunoprecipitation of FLAG was done. Immunoprecipitated samples were used for Western blot (WB) using phospho-β-catenin (92 kDa) and FLAG antibody (92 kDa; β-catenin expression). The average expression of phospho-β-catenin was shown by graphical representation after three experiments and FLAG was taken as internal control. Error bars show mean values ± SE, *p < 0.01. **c** β-catenin KO cells were transfected with FLAG-wild-type (Wt), FLAG-phospho-mutant (S552A) β-catenin plasmids in presence or absence of either AKT inhibitor, or co-transfected with constitutive phospho-mimic AKT1 (HA-AKT1DD) plasmid. All samples were infected with parasite for 12 h, including cells of Wt-β-catenin with HA-AKT1DD, and total cell lysates were used for the Western blot using SAG1 (36 kDa) antibody. The average expression of SAG1 was shown by graphical representation after three experiments and β-actin was taken as internal control. Error bars show mean values ± SE, ✶*p* < 0.01. **d** Caco2 cells, seeded at ~60% confluency, were transfected with GFP-wild-type-β-catenin (Wt), and 12 h of transfection, infected with *T. gondii*. At indicated times, cells were stained with the DNA-binding dye, DAPI, to locate the nucleus (blue), and observed under microscope. ‘Only cell’ indicate uninfected cells. Scale bar = 10 µm. In the Western blots, cells were transfected with (**e**) FLAG-wild-type (Wt)-β-catenin or (**f**) FLAG-phospho-mutant-β-catenin, or (**h**) FLAG-phospho-constitutive-β-catenin plasmid, and infected with *T. gondii*. At indicated times, nucleus and cytoplasm were purified and immunoblot was done with FLAG antibody. The purity of the nucleus and cytoplasm was ascertained using histone (17 kDa) and α-tublin (52 kDa) antibody respectively. Caco2 cells were transfected with (**g**) GFP-phospho-mutant β-catenin (**i**) GFP-constitutive β-catenin plasmid, no infection, (**j**) GFP-constitutive β-catenin plasmid, followed by 24 h post-infection. Cells were stained with DAPI for nucleus and localization of GFP protein was observed under fluorescence microscope. Scale bar = 10 µm. β-catenin-KO cells were transfected with FLAG-Wt-β-catenin, or FLAG-phospho-mutant (S552A), or FLAG-phospho-mimic-β-catenin (S552D) and *T. gondii* growth was measured after infection by (**k**) SAG1 antibody (36 kDa) and (**l**) qPCR using ITS-1 primer. The mean band intensity of three separate blots was presented in bar diagram. In only cells, backbone pcDNA3.1 was transfected. Error bars show mean values ± SE of three separate experiments, *p < 0.001. **m** β-catenin-KO cells were transfected with FLAG-Wt-β-catenin, or FLAG-phospho-mutant (S552A), or FLAG-phospho-mimic-β-catenin (S552D) and after infection with mCherry expressing *T. gondii* and at the indicated times, stained for nucleus (DAPI, blue) and visualized by confocal microscopy

Phospho-AKT mediated β-catenin-TCF4 complex acts as a transcription factor for several gene expressions^3,34^. Furthermore, parasite growth was supported by AKT-Ser473 phosphorylation (Fig. 2a). To determine the essentiality of AKT in β-catenin phosphorylation, we expressed FLAG-tagged Wt or phospho-mutant S552A-β-catenin plasmid in β-catenin KO cells. Wt-β-catenin expressing cells showed β-catenin phosphorylation (lane 2, Fig. 2b), and parasite growth was also detected (lane 2, Fig. 2c). Both phospho-β-catenin (lane 3, Fig. 2b) and parasite growth (lane 3, Fig. 2c) were absent in S552A mutant-β-catenin expressing cells after infection. In presence of constitutively active AKT1 (HA-AKT1DD), Wt-β-catenin was phosphorylated even in absence of infection, as evidenced by high-band intensity (lane 4, Fig. 2b). However, *T. gondii* multiplied faster in presence of AKT1DD (lane 4, Fig. 2c). Both β-catenin phosphorylation (lane 5, Fig. 2b) and parasite growth (lane 5, Fig. 2c) were abrogated in presence of AKT inhibitor-IV.

### Phospho-β-catenin is critical for parasite replication

To check the cellular distribution of β-catenin upon infection, GFP fluorescence intensity was found to be higher in cytoplasm than in the nucleus at 12 h post-infection; however, 18 h onwards, GFP-β-catenin migrated to nucleus, which was essentially complete at 24 h post-infection (Fig. 2d). To determine if β-catenin phosphorylation is important for nuclear translocation, results showed that Wt-β-catenin translocated into nucleus following infection (Fig. 2e). In contrast, phospho-mutant-β-catenin failed to move into nucleus (Fig. 2f), as all GFP-S552A-β-catenin was wedged outside of nucleus (Fig. 2g). Phospho-mimic-β-catenin translocated even in uninfected cells (Figs. 2h, i), although the amount was higher in infected cells nuclei (Figs. 2h–j).

We observed that β-catenin phosphorylation at S552 is indispensable for optimum parasite multiplication. Wt-β-catenin supported robust parasite replication, which was significantly reduced in presence of phospho-mutant β-catenin. Reciprocally, constitutively active phospho-mimic strongly restored parasite growth (Figs. 2k, l). Microscopic study showed that most of the parasites were unable to internalize and incompetent to form PVs in presence of phospho-mutant expressing β-catenin cells. However, the intracellular parasites load and formation of PVs were fully restored in presence of phospho-mimic β-catenin (Fig. 2m, Supplementary Fig. S1b).

### β-catenin-TCF binding site is present at IRF3 promoter

**Fig. 3:**
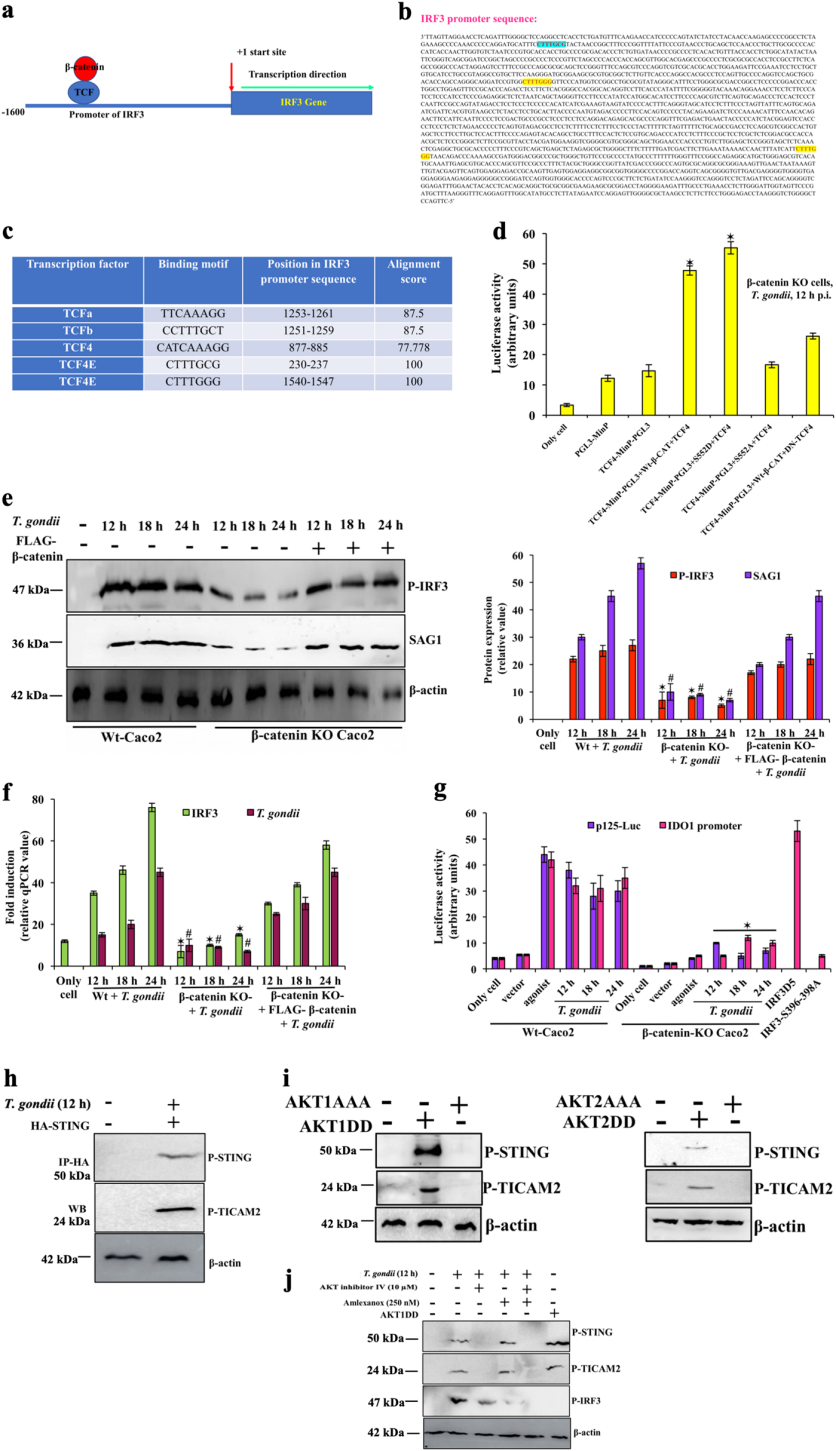
*T. gondii* infection recruits TCF to IRF3 promoter, and maintain its functional activity. **a** This is the hypothetical model where TCF could bind at the promoter region of IRF3 and regulates the transcription of IRF3. **b** IRF3 promoter sequence has two consensus sequences, CTTTGGG, and CTTTGCG where TCF prefers to bind. **c** There were several isomers of TCF with their binding site, present on IRF3 promoter site and the alignment score was calculated for individual isomer. **d** TCF4-MinP-PGL3 is (CTTTGGG/CTTTGCG) TCF binding site on IRF3 promoter with minimal promoter in pGL3 luciferase plasmid was cloned. TCF4-MinP-PGL3 was transfected with Wt-β-catenin + TCF4 plasmids, or phopsho-mimic-β-catenin (S552D) + TCF4 plasmids, or phopsho-mutant-β-catenin (S552A) + TCF4 plasmids, or Wt-β-catenin + dominant-negative (DN)-TCF4 plasmids in β-catenin KO cells. pGL3-minimal promoter (PGL3-MinP) luciferase plasmid was taken as negative control and pCMV-Renilla-Luc plasmid was transfected in each condition. At 12 h post infection, cells were harvested for dual luciferase assay of TCF4-MinP-PGL3. The firefly luciferase units were normalized against the Renilla units and expressed in percentage. Values were averaged from four similar experiments, with error bars shown, *p < 0.001. Wild-type Caco2 cells (Wt), only β-catenin-KO cells and β-catenin-KO cells with FLAG-β-catenin plasmid were infected with *T. gondii* and (**e**) phosphorylation of IRF3 and parasite growth were determined using phospho-IRF3(S396) (47 kDa), and SAG1 (36 kDa) antibody respectively and data of mean ± SE of three individual experiment was represented by bar diagram. *, ^#^*p* < 0.01. **f** mRNA of IRF3 and *T. gondii* was quantified by real-time PCR. Data was represented by bar diagram after having average ± SE of three individual experiment. *, ^#^*p* < 0.01. **g** p125-luc (IFN-β promoter activity) in presence of IFN-β (agonist) and IDO1 promoter activity in presence of IFN-γ (agonist) was tested. In separate wells, p125-Luc or IDO1 reporter plasmid were transfected in Wt-Caco2 and β-catenin-KO cells and parasite infection was done. IDO1 promoter activity was also checked in presence of phospho-mimic (IRF3D5), and phospho-mutant IRF3 (IRF3-S396-398A) constructs. Only cell is without plasmid, and Vector (pGL3 basic) was used as control. pCMV-Renilla Luc plasmid was transfected in each sample, and used as internal control. At indicated time post-infection, cells were harvested for dual luciferase assay. The firefly luciferase units were normalized against the Renilla units and expressed in percentage. Average values of three experiments, with error bars show mean values ± SE, *p < 0.01. **h** Un-transfected-uninfected cells and cells transfected with HA-STING, followed by 12 h of *T*. *gondii* infection were immunoprecipitated using HA antibody. After immunoprecipitation, all samples were separately Western blotted (WB) using phospho-STING (50 kDa), and phospho-TICAM2 antibody (24 kDa). **i** Caco2 cells were transfected with phospho-constitutive AKT1DD/AKT2DD or phospho-mutant AKT1AAA/AKT2AAA. After 8 h of transfection, fresh media was added. 24 h post-transfection, cell lysates were immunoblotted using P-STING(S366), and P-TICAM2 antibody. **j** Caco2 cells were infected with *T. gondii* in presence or absence of either AKT inhibitor, or TBK inhibitor. One well of Caco2 cells were only transfected with constitutive phospho-mimic AKT1 (HA-AKT1DD) plasmid without any infection or inhibitor. All samples were used for the immunoblot using phospho-STING, phospho-TICAM2, and phospho-IRF3(Serine antibody). In **i**, and **j**, we did not get dimer of STING. This blot is the average representation of three separate experiments

We hypothesized that activated β-catenin-TCF complex may facilitate *T. gondii* replication by inducing IRF3 gene expression (Fig. 3a). CLUSTALW multiple sequence alignment indeed revealed multiple TCF-binding sequences at the IRF3 promoter (ENSG00000126456; Fig. 3b), and the binding motif CTTTGGG/CTTTGCG of IRF3 promoter showed an alignment score of 100 (Fig. 3c). To test the functionality of the sites, we constructed the TCF4-MinP-pGL3 luciferase reporter vector. In β-catenin KO cells, CTTTGGG/CTTTGCG-promoter site dependent IRF3 transcription was abrogated, as there was no luciferase activity from TCF4-MinP-pGL3 alone, which clearly indicated that in absence of β-catenin, cellular TCF4 could not bind at IRF3 promoter sequences (Fig. 3d). High promoter activity of TCF4-MinP-pGL3 was observed during transient transfection of Wt-β-catenin and TCF4, but not when S552A-mutant-β-catenin and TCF4 were used. The phospho-mimic β-catenin(S552D) induced the highest luciferase activity. Lastly, TCF4 dominant-negative mutant suppressed CTTTGGG/CTTTGCG-dependent IRF3 transcription (Fig. 3d). Overall, these results demonstrate that β-catenin is essential for IRF3 gene expression.

### Functional activity of IRF3 is dependent on β-catenin

We measured total IRF3 mRNA and phospho-IRF3 in Wt-Caco2 cells, which were increased along with augmented parasite replication (Figs. 3e, f). However, in β-catenin KO Caco2 cells, in contrast, both mRNA level and phospho-IRF3 along with parasite growth were highly reduced, but were essentially fully reinstated when recombinant FLAG-β-catenin was expressed in β-catenin KO cells (Figs. 3e, f), suggesting the significance of β-catenin in IRF3 activation.

To reinforce our hypothesis, we measured promoter activity of IFN-β, which is IRF3-dependent. *T. gondii* infection induced strong luciferase activity of p125-luc (a reporter plasmid with IFN-β promoter) at all-time points. We also found strong promoter activity of another ISG candidate, IDO1, in infection, but significant loss of both p125-luc and IDO1 promoter activity was observed in β-catenin KO cells (Fig. 3g). We conclude that transcription of both IFN-β and IDO1 promoters were dependent on functional IRF3. As functional IRF3 activity is dependent on β-catenin, therefore transcription of IDO1 were also inhibited in absence of β-catenin.

### Phosphorylation of STING, and TICAM2 is AKT dependent

We recently reported an essential role of STING in parasite growth^10^. Here we showed an adaptor molecule TICAM2 binds with STING for optimum infection. HA-STING was expressed by transfection, immunoprecipitated with HA antibody, and immunoblotting was done separately with phospho-STING, and phospho-TICAM2 antibodies. Neither phospho-STING, nor phospho-TICAM2 were detected in uninfected cells, but at 12 h post-infection, immunoprecipitated sample expressed phsopho-STING, and phospho-TICAM2 (Fig. 3h). Transient transfection of AKT1DD(308D473D), or AKT2DD initiated phosphorylation of cellular STING and TICAM2 even in absence of infection, which was inhibited by dominant-negative AKT1AAA(K179A/T308A/S473A) or AKT2AAA (Fig. 3i). These results together concluded, AKT mediated phospho-STING and phospho-TICAM2 formed a heterodimer during infection for downstream activation. To distinguish the role of AKT and TBK1 in phosphorylation of STING, TICAM2, and IRF3, we used AKT inhibitor (AKT inhibitor IV) and TBK1 inhibitor (amlexanox) (Fig. 3j). Phosphorylation of STING, and TICAM2 were inhibited, but not IRF3 phosphorylation by AKT inhibitor IV after infection. But, in presence of amlexanox which prevented cellular TBK expression, STING and TICAM2 were phosphorylated in infection, but IRF3 phosphorylation was inhibited (Fig. 3j). In presence of both the AKT and TBK inhibitors, phosphorylation of all three molecules was abrogated. Upon transient expression of constitutive AKT1DD, the intensity of phospho-STING, and phospho-TICAM2 bands was augmented significantly, even in uninfected cells but, there was no effect of IRF3 phosphorylation. Based on these results, we conclude that *T. gondii* infection induces TBK-independent AKT-mediated phosphorylation of STING, and TICAM2, whereas IRF3 phosphorylation is completely dependent on TBK. These results support a cardinal role of AKT in β-catenin activation (Fig. 2b), as well as STING-TICAM2 dependent downstream IRF3 signalling (Figs. 3i, j).

### β-catenin-IRF3 dependent IDO1 negatively regulates *T. gondii* growth

**Fig. 4:**
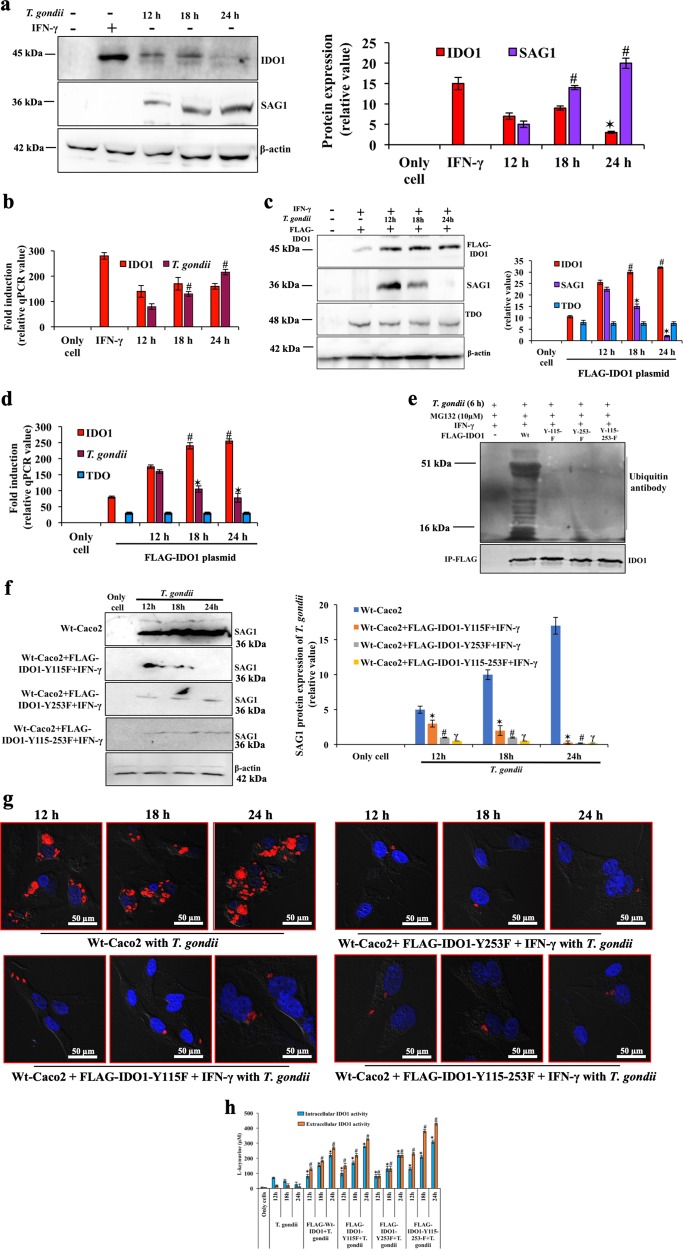
Functional tyrosine-mutant IDO1 inhibits multiplication of *T. gondii*. **a** Infection and followed by immunoblotting was performed with IDO1 (45 kDa), SAG1 (36 kDa) antibody in Caco2 cells. The mean ± SE of three repeats were shown by bar diagram (right panel). IFN-γ was taken as an IDO1 activator. **b** mRNA expression of IDO1, and *T. gondii* was determined by qPCR after infection. In both, IFN-γ was taken as IDO1 agonist. **c, d** Cells were over-expressed with FLAG-IDO1 plasmid (4 µg in 4 cm^2^ well) and immunoblot was done using FLAG (45 kDa), SAG1 (36 kDa), TDO (48 kDa), and β-actin antibody (42 kDa). Three repeats of protein and mRNA expression was exhibited by bar diagram using β-actin as internal control. Data represents mean ± SE of three repeats. * and ^#^ represent statistically significant (*p* < 0.05), comparison was made relative to 12 h infected cells. **e** Cells were transfected with the following plasmids (0.5 µg in 4 cm^2^ well): FLAG-wild-type IDO1 (Wt), tyrosine single/double mutant (FLAG-Y-115F, FLAG-Y-253-F, or FLAG-Y-115-253-F) in the presence of IFN-γ, and MG132. Total cell-lysates were subjected to immunoprecipitation using FLAG antibody, followed by the immunoblot was done using ubiquitin antibody. The higher relative mobility of the bands indicates multiple tyrosine phosphorylation. Cells were transfected with the following plasmids (0.5 µg in 4 cm^2^ well): FLAG-tyrosine single/double mutant (FLAG-Y-115F, FLAG-Y-253-F, or FLAG-Y-115-253-F) in the presence of IFN-γ. After 8 h of transfection, untransfected and transfected cells were infected with parasite. **f** Total cell-lysates were used for immunoblot using SAG1 antibody (36 kDa). Bar diagram represents mean ± SE of three repeats of SAG1 band. *, ^#^, ^*γ*^ represent statistically significant (*p* < 0.05), comparison was made relative to untransfected-infected cells of each time-point. **g** Cells were transfected with the above-mentioned plasmids and infection was done by mCherry expressing *T. gondii* RH, and at the indicated times, slides were stained for nucleus (DAPI, blue) and visualized by confocal microscopy. **h** In similar condition, infected cells and cell supernatants were collected for measuring functional enzymatic activity of IDO1. Kynurenine production was assayed by photometric assay. Bar diagram represents mean ± SE of three separate experiment. *, and ^#^ represent statistically significant (*p* < 0.05), comparison was made relative to only untransfected-infected cells of each time-point

As mentioned earlier, IDO1 activity catalyzes tryptophan degradation, which might suppress parasite growth. We observed, IDO1 promoter activity is supported by β-catenin during infection (Fig. 3g). IDO1 transcription is also directly controlled by active IRF3. Constitutive phospho-mimic-IRF3D5 alone promoted IDO1 transcription which relied on serine phosphorylation, since the IRF3-S-396-398A mutant-construct abolished IDO1 luciferase activity (Fig. 3g). When IDO1 expression was induced by IFN-γ, the expression of IDO1 mRNA (Fig. 4b) and protein levels (Fig. 4a) were discordant in *T. gondii*-infected cells: IDO1 protein levels were dramatically reduced (Fig. 4a), while mRNA levels were slightly increased (Fig. 4b). To test our hypothesis that the decrease in IDO1 protein levels was instrumental in assisting *T. gondii* replication, we overexpressed IDO1 in cells by transfecting FLAG-IDO1 plasmid. Higher amounts of IDO1 in cells indeed stunted *T. gondii* growth as both SAG1 (Fig. 4c) and ITS-1 mRNA levels were reduced (Fig. 4d). Transfection with empty vector p-EF-BOS did not have any effect on parasite growth (Supplementary Fig. S2a). Tryptophan was catabolized into kynurenine with overexpression of Wt-IDO1 (bars 5–7, Fig. 4h, helped in limiting parasite growth (second panel, Fig. 4c). In these experiments, we did not get any visible expression of IDO2 (data not shown) during the entire length of infection, and the level of TDO was also unchanged both in uninfected and infected cells (Fig. 4c, d).

IDO1 has been reported to possess two tyrosine (Y) residues within two distinct putative immunoreceptor tyrosine-based inhibitory motifs, VPY-115-CEL and LLY-253-EGV, which are important for UPS recognition^35^. Thus, to explore the mechanism of IDO1 protein depletion, we investigated a possible role of the UPS. Cells were transfected with FLAG-IDO1, or tyrosine single mutant FLAG-Y-115F, or FLAG-Y-253-F, or double mutant FLAG-Y-115-253F, in presence of MG132, and infection was done. At 6 h post-infection, cell lysates were immunoprecipitated with anti-FLAG antibody, and immunoblotted with anti-ubiquitin antibody. We found that Wt-IDO1 was ubiquitinated, whereas the single and double mutants were not (Fig. 4e). These results decipher that IDO1 is a substrate of UPS and *T. gondii* degraded IDO1 through its tyrosine phosphorylation for smooth replication. Thus, tyrosine mutant IDO1 expressing cells impaired *T. gondii* replication (Fig. 4f), because most of the parasites were unable to internalize (Fig. 4g, Supplementary Fig. S1c). Parasites that entered inside tyrosine mutant-IDO1 expressing cells, were not able to form PVs in process of infection (Supplementary Fig. S1c). IDO1 was stable and active in absence of its phosphorylation, and increased tryptophan catabolism into kynurenine (Fig. 4h). This results suggest that parasite growth was stunted in tyrosine mutant IDO1 expressing cells (Fig. 4g), because the tyrosine metabolite, kynurenine inhibited its growth.

### Tryptophan catabolism decides the fate of *T. gondii* by regulating β-catenin

**Fig. 5:**
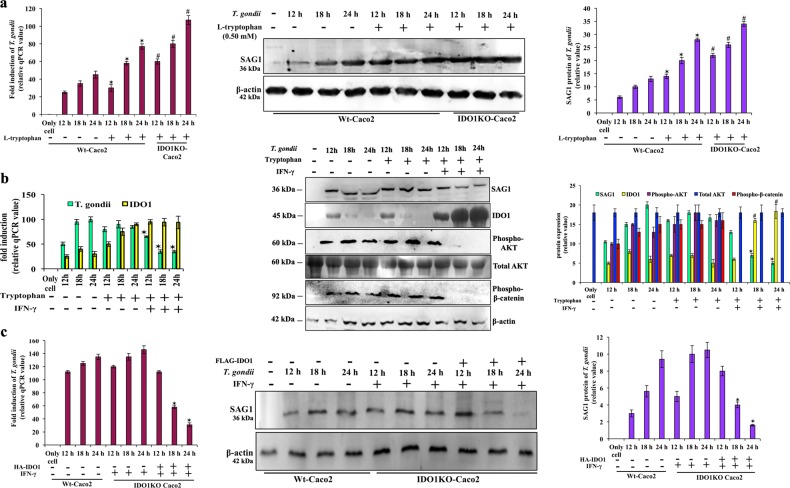
Tryptophan catabolites decide the fate of *T. gondii* replication. **a** Wild type (Wt), and IDO1-knock-out (KO) cells were incubated with or without tryptophan and *T. gondii* growth was checked by qPCR (ITS-1), and immunoblot (SAG1), and average ± SE of intensity of band of three similar blots were denoted in bar diagram. *p < 0.01 and ^#^*p* < 0.01, evaluation was made relative to corresponding time-point, infected with only parasite. **b** Caco2 cells were infected with *T. gondii* in absence and presence of tryptophan, with or without IFN-γ, and growth of parasite and IDO1 expression were determined by qPCR. Parasite growth (SAG1), IDO1, phopsho-AKT, total AKT and phospho-β-catenin expression were checked by immnoblotting and the average of three repeats were expressed in bar diagram with error bars using β-actin as internal control. *p < 0.05 **c** Similarly, Wt, IDO1KO, IDO1 restored by HA-IDO1 plasmid in IDO1KO cells were infected with parasite in presence or absence of IFN-γ. ITS-1 expression was determined by qPCR and SAG1 expression was determined by immunoblot. The mean ± SE of three repeats were expressed in bar diagram with error bars using β-actin as internal control. *p < 0.001

To delineate the role of IDO1 in β-catenin expression for controlling *T. gondii* growth, we explored its function in greater detail by manipulating tryptophan as well as IDO1 levels. Firstly, we added L-tryptophan to Wt and IDO1KO Caco2 cells and measured parasite growth. While exogenous tryptophan nearly doubled the parasite growth in Wt cells (lane 5-7, Fig. 5a), the effect was even significantly higher in IDO1KO cells (lane 8-10, Fig. 5a). Parasite growth was further augmented in IDO1KO cells in presence of L-tryptophan, compared to IDO1KO cells without tryptophan (Supplementary Figure S2b). Tryptophan was converted to higher amounts of melatonin in IDO1KO cells which were externally supplemented with tryptophan during infection (Supplementary Figure S2c). These results suggest that *T. gondii* catabolizes tryptophan into its final derivative, melatonin (Supplementary Figure S2c, d) by inhibiting IDO1 activity (lane 2–7, second panel, Fig. 5b). Since IDO1 was degraded by UPS, it leads to concomitant decrease in both extracellular and intracellular kynurenine (Supplementary Figure S2e, f) which promoted optimal parasite multiplication (Fig. 5a). To explore further, we measured the fate of phospho-AKT which is a central molecule in this study. We found stable phospho-AKT during infection (lane 2–4, third panel, Fig. 5b), and this phospho-AKT was further augmented in presence of supplementary tryptophan (lane 5–7, third panel, Fig. 5b), because increased melatonin (Supplementary Figure S2d) was able to maintain cell alive. However, tryptophan in presence of IFN-γ was catabolized into kynurenine (Supplementary Figure S2e, f) by amplified IDO1 activity (lane 8–10, second panel, Fig. 5b), which inhibited *T. gondii* replication (lane 8–10, first panel, Fig. 5b). The increased IDO1 activity and resultant decreased parasite growth was mediated through depletion of phospho-AKT (lane 8–10, third panel, Fig. 5b) and total AKT remained same in all conditions (fourth panel, Fig. 5b). β-catenin phosphorylation (lane 2–7, fifth panel, Fig. 5b) was further correlated with enhanced functional AKT activity. Thus, we conclude externally supplemented tryptophan in presence of IFN-γ augmented cellular IDO1 which negatively regulated activity of both AKT and β-catenin to prevent parasite infection (lane 8–10, first panel, Fig. 5b).

Lastly, we treated Wt and IDO1KO cells with IFN-γ, a known inducer of IDO1. We previously reported that IFN-γ suppresses *T. gondii* replication by inducing T helper-1 (Th1) response^10^. We have evidenced here that IFN-γ alone cannot impede parasite growth in absence of IDO1 (lane 5–7, Fig. 5c), but does so to a significant level when IDO1 is overexpressed (lane 8–10, Fig. 5c).

### Melatonin dependent host cell-survival supports robust *T. gondii* multiplication

**Fig. 6:**
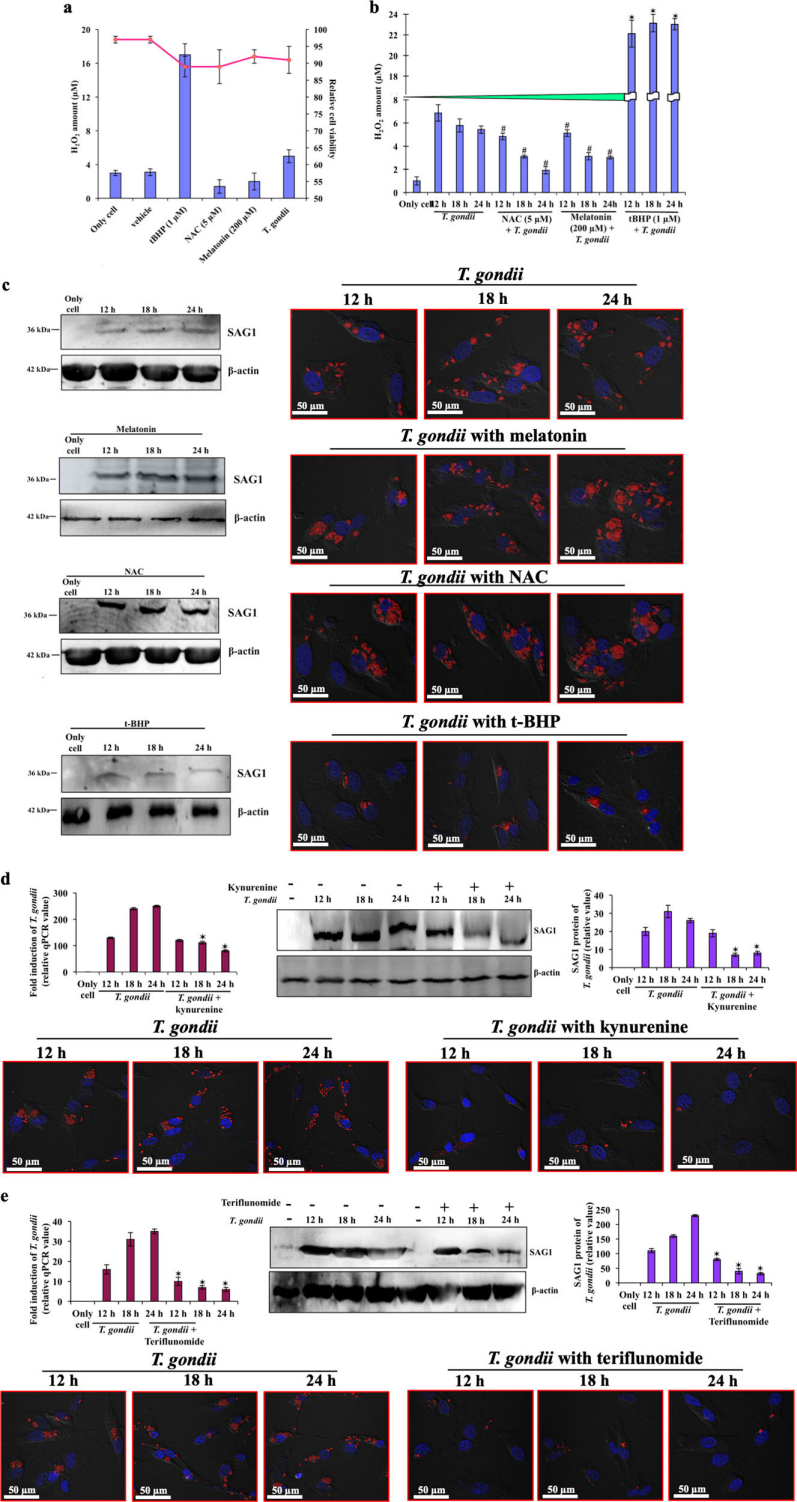
ROS impedes *T. gondii* replication, followed by small molecule treatment. **a** Cells were incubated with tBHP, or NAC, or melatonin or infected with parasite (MOI 2) and released H_2_O_2_ from cells were measured spectrophotometrically, and viability of cells were determined by trypan blue. **b** Cells were incubated with NAC, or melatonin, or tBHP, followed by infection with parasite. At indicated time points, released H_2_O_2_ was measured. Data showed average values ± SE of three repeated experiments. ✶ and # represent statistically significant (*p* < 0.05), comparison was made relative to cells infected with parasite only. **c** Infected cells were treated with either NAC, or melatonin, or tBHP and immunoblot was done using SAG1 antibody to check the parasite growth. To calculate the number of intracellular parasites and their ability to develop PVs, confocal microscopy was done using mCherry expressing parasites in similar conditions. *T. gondii* infected cells were treated with **d** kynurenine and **e** teriflunomide. The growth of parasites was checked by both qPCR and the immunoblot as well as by confocal microscopy. Bar diagram denotes the mean ± SE of four independent experiments. * highlights statistically significant (*p* < 0.01), each time point compared with same time point of infected cells without any treatment

Optimal *T. gondii* replication has been shown to require host cell viability^36,37^. We, therefore, investigated the cell survival mechanism in context of tryptophan catabolism and parasite replication. We measured ROS levels after infection and tested its impact by stimulating ROS with *tert*-butyl hydroperoxide (tBHP) or by inhibiting it with *N*-Acetyl-L-cysteine (NAC) or melatonin treatment (Fig. 6a). The concentration of tBHP or NAC and melatonin, or the multiplicity of infection (MOI) of *T. gondii* used here did not induce significant cytotoxicity of cells, as >90% cells were viable (Fig. 6a). We also found that *T. gondii* reduced the H_2_O_2_ production in course of infection. The H_2_O_2_ levels were further reduced in NAC or melatonin treated infected cells (Fig. 6b), and this inhibition of ROS by both these molecules augmented parasite growth as observed by both immunoblot and microscopic studies (Fig. 6c, Supplementary Fig. S1d). Conversely, in presence of tBHP, H_2_O_2_ level was increased in infection (Fig. 6b), and elevated ROS suppressed parasite growth, as evidenced by the scarcity of intracellular PVs during infection (fourth panel, Fig. 6c, Supplementary Fig. S1d).

### Kynurenine or teriflunomide prevents *T. gondii* growth

As discussed earlier, functional IDO1 activity leads to in annulment of parasite growth. To study its mechanism further, we treated infected cells with kynurenine or its analogue teriflunomide, and measured parasite growth. At both mRNA and protein levels, kynurenine (Fig. 6d) and teriflunomide (Fig. 6e) reduced *T. gondii* growth significantly. Microscopic analysis showed that parasites did not enter significantly after kynurenine or teriflunomide treatment in wild-type Caco2 cells. Once again, few parasites that were seen inside were not competent to produce PVs (Figs. 6d, e, Supplementary Fig. S1d).

**Fig. 7:**
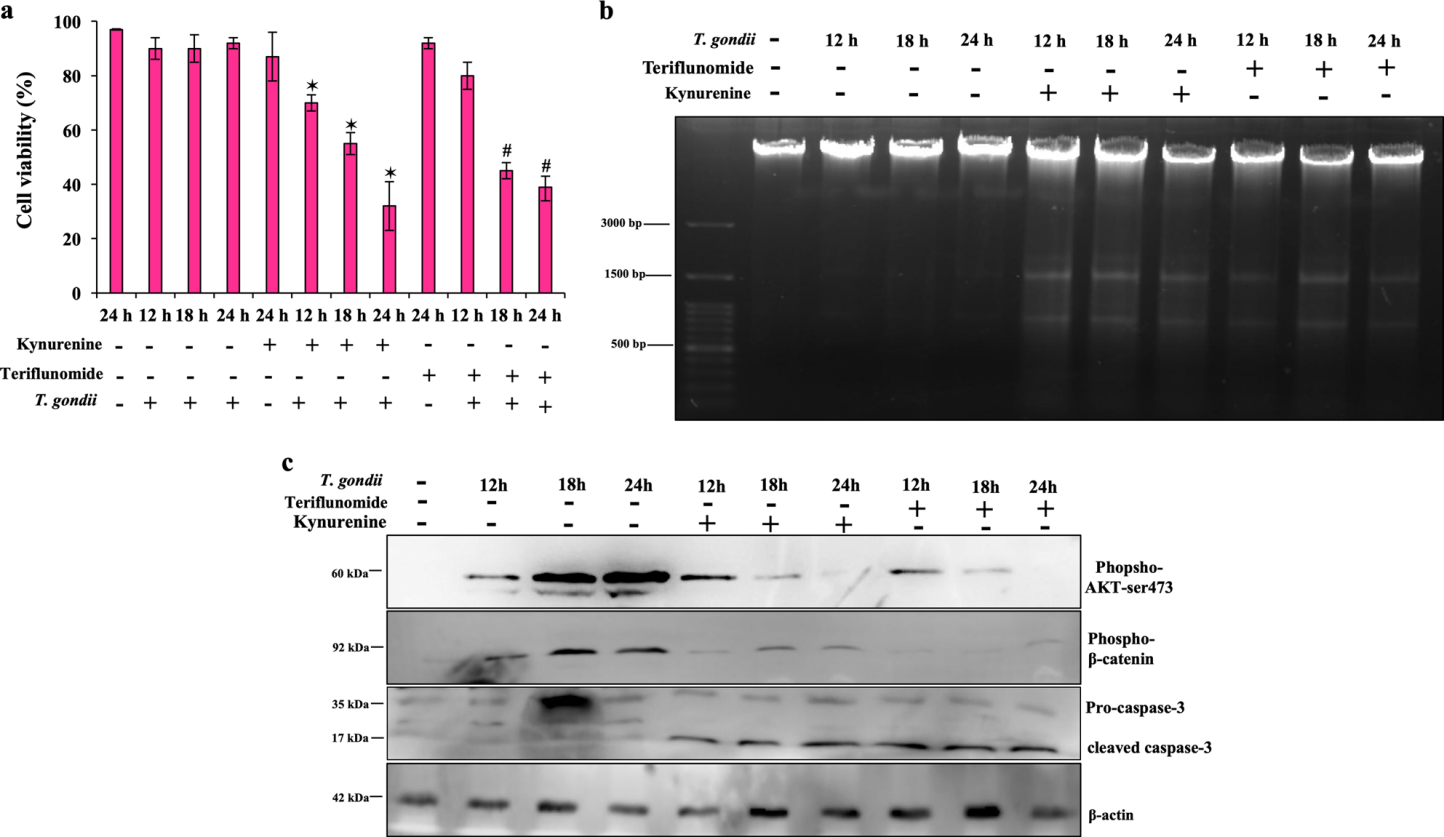
Kynurenine and teriflunomide treatment induces apoptotic cell death. **a** Infected cells were treated with either kynurenine or teriflunomide, and at indicated time points, cell viability was measured by trypan blue. Error bars show mean values ± SE of three repeated experiments. * and ^#^ represent statistically significant (*p* < 0.05), comparison was made relative to cell, only infected with parasite at the corresponding time point. **b** Genomic DNA was isolated from *T. gondii* infected cells, which were treated with or without kynurenine or teriflunomide separately and pattern of DNA fragmentation was shown by agarose gel electrophoresis. **c** *T. gondii* infected cells, which were treated with or without kynurenine or teriflunomide separately, and total cell-lysates at indicated time was collected for immunoblotting using phospho-AKT (60 kDa), phospho-β-catenin (92 kDa) and caspase-3 antibody (35 kDa, 17 kDa). The blot is the representation of three separate experiments

As before, *T. gondii* infection did not affect cell viability, as > 90% cells remained viable at all time points of infection (Fig. 7a). Based on our microscopic observation, kynurenine (0.50 mM) or teriflunomide (0.50 mM) alone halted the speed of cell division, as these molecules promote cytostasis, but could not induce appreciable cytotoxicity in healthy cells after 24 h (Fig. 7a). Nonetheless, treatment of infected cells with kynurenine or teriflunomide had significant effect on cell death (Fig. 7a). We isolated genomic DNA from infected cells, and checked DNA breakage pattern, characteristics of apoptosis. Wt-Caco2 cells, infected with parasite, did not undergo any DNA breakage, and cells were also viable; however, after drug treatments, infected cells experienced apoptotic death, and DNA was fragmented (Fig. 7b). We also tested the mechanism of apoptosis in infected cells after drug treatment. Early death of cells inhibited parasite growth, which is consistent with our findings that kynurenine or teriflunomide treatment promoted depletion of phospho-AKT levels, thus infected cells were progressed towards apoptosis (lane 5–7, lane 8–10, first panel, Fig. 7c). Depleted AKT activity circumvented β-catenin phosphorylation (lane 5–7, lane 8–10, second panel) which is the most critical molecule to maintain cell viability which in turn required to support the optimal parasite replication. To delineate further the mechanism of apoptosis promoted by kynurenine and teriflunomide, infected cells, treated with these small molecules, were analysed for caspase activity. Both kynurenine and teriflunomide which stimulated cleavage of pro-caspase-3 (35 kDa) to active caspase-3 (17 kDa) in *T. gondii*-infected cells, were ultimately responsible for cellular alternations associated with apoptosis (third panel, Fig. 7c).

## Discussion

Here we have uncovered a novel function of the phospho-β-catenin-TCF protein complex, in regulating IRF3 transcription to enhance host cell viability through β-catenin phosphorylation, facilitating sustained parasite growth. Inhibition of β-catenin depleted parasite growth as transcription and phosphorylation of IRF3 was halted. This is the first example where the promicrobial function of β-catenin-TCF4 complex regulates IRF3-dependent innate immunity for the benefit of parasite multiplication (Supplementary Figure S3a). Here, AKT is the key factor which phosphorylated β-catenin, STING, and TICAM2 for downstream IRF3 activation by TBK (Supplementary Figure S3b). Thus, AKT is the universal molecule that bridges functional activity of β-catenin with STING-TICAM2-IRF3-mediated innate immunity. It remains to be seen whether this new paradigm is true for other intracellular protozoa as well. It is interesting to note that several IRF3-depedent ISG molecules supported parasite growth^10,38^. As a pro-parasitic molecule, IRF3 controlled expression of IDO1, one of the major ISGs, but their function is conflicting. IDO1 negatively regulated parasite growth, as phosphorylation dependent IDO1 degradation augmented parasite replication. Earlier, we witnessed that several ISGs such as ISG-54, ISG-56 are directly controlled by IRF3 which promoted ISGs transcription in an IFN-I independent fashion^10^. Here, we have noted that phospho-IRF3 has ability to encourage direct IDO1 transcription (Fig. 3g). Phosphorylation of AKT, β-catenin, STING, and IRF3 at Ser residue and IDO1 phosphorylation at the Tyr residue benefited parasite growth, but their own fates are discordant. Serine phosphorylation provided stability to those molecules, whereas tyrosine phosphorylation directed IDO1 degradation. Although, we found that Ser mutant of β-catenin (Fig. 2k–m) as well as Tyr mutant of IDO1 (Fig. 4f, g) provided protection against infection. These phospho-mutation of different molecules inhibited parasite growth by abrogating parasites internalization and inhibiting PVs formation (Figs. 2m, 4g, Supplementary Fig. S1b, c). Ser mutation of β-catenin impaired pro-parasitic IRF3 activation, and Tyr mutation escaped UPS-mediated IDO1 degradation. This stable tyrosine-mutant IDO1 provided protection against infection through kynurenine catabolism (Fig. 4h). Tryptophan is an essential amino acid, and its IDO1-reliant catabolism is a key factor that controls the growth of several intracellular pathogens^39–42^.

Protozoan intracellular parasites like *T. gondii* are tryptophan auxotrophs^43^ and there are evidences of a Jekyll and Hyde role of IDO1 in *T. gondii* infection. The earliest reports demonstrated that inhibition of several parasites growth is IFN-γ-IDO dependent^27,44^. More recently, augmented IDO1 activity was shown to diminish the progression of *Leishmania major* infection^39^. In contradiction, there were reports where IDO1 depletion attenuated *T. gondii* replication in lungs^45^. This divergent role of IDO1 suggested that it is not a lone regulator that acts as an antimicrobial or promicrobial factor. Catabolic fate of tryptophan and regulation of functional IDO1 are also important to determine the fate of parasite infection (Supplementary Figure S3c).

Our results suggest that over-expressed IDO1 diminished cellular kinase activity as phsopho-AKT level was low with higher IDO1 (Fig. 5b). As kinase is the signature molecule for phosphorylation of different cellular proteins, thus, chances of phosphorylation of over-expressed IDO1 were reduced, and un-phosphorylated IDO1 remained stable to prevent infection (Figs. 4c, 5b). Consequently, AKT related phospho-β-catenin-TCF mediated downstream signalling as well as STING-TICAM2-IRF3 signalling were also crippled, leading to cell death and limiting parasite growth (Supplementary Figure S3d). We conclude, therefore, tyrosine phosphorylation mediated proteasomal degradation of IDO1 maintains cell survival which is essential for *T. gondii* multiplication.

In absence of IDO1, tryptophan was catabolized into melatonin, suppressed ROS to maintain healthy cells and sustained parasite multiplication. In presence of IFN-γ, IDO1 activity was stabilized and tryptophan was catabolized into its first stable derivative, kynurenine (Supplementary Figure S3d). Kynurenine also acts as an inducer of apoptosis in *T. gondii-*infected cells, inhibiting parasitic growth. However, kynurenine is a physiological metabolite, and its use as an anti-parasitic drug might have untoward consequences on human health. Thus, we opted for an alternative approach to modify kynurenine pathway, which would shape the balance of kynurenine towards neuroprotection. Currently, there are several analogues of kynurenine, either commercially available or under clinical trials. Teriflunomide, for example, increases the synthesis of kynurenines in brain, and offers protection from seizures^46,47^. Interestingly, toxoplasmosis has been suspected to be a risk factor for various neurological disorders^48,49^. Earlier, niacin was used as a drug because it is final product of kynurenine pathway, but niacin has feed-back effects, as higher amounts of niacin exhausts the enzymatic activity of IDO1^50^.

Kynurenine inhibited AKT pathway, directed to caspase-3 dependent cellular apoptosis for retardation of parasite growth. Kynurenine also activates Wnt pathway but AKT phosphorylation was crippled, so β-catenin activity was also inhibited^51^. Therefore, pro-parasitic IRF3 transcription in nucleus would be halted as well. Recent studies showed that kynurenine pathway induces caspase-dependent apoptosis, but NAC prevents apoptosis by inhibiting ROS^52^. Thus, sustained infection occurred through depletion of intracellular ROS. Here, we propose that induction of the kynurenine pathway and/or controlling the systemic tryptophan concentrations by stimulation of immune cells or by diet may constitute novel and effective strategies to treat parasite infection.

## Materials and methods

### Reagents

The following chemicals were obtained from commercial sources, and were used as follows:

(i) The Wnt agonist, AMBMP hydrochloride (Tocris), used as positive control for β-catenin activation, was reconstituted into 10 mM of final concentration in 2 µl DMSO which did not exhibit any cell toxicity (not shown). (ii) AKT Inhibitor IV (Sigma) was used at the concentration of 10 μM in DMSO. (iii) For 250 nM of amlexanox (Tocris), 0.12 µl of 1 mM of stock solution (1 mg/3.35 ml DMSO) was used in 500 µl cell culture. (iv) L-tryptophan (Sigma) was reconstituted at final concentration of 0.50 mM in H_2_O. (v) Human IFN-γ (Sigma, 1000 units/ml) was used in 1 ml of cell culture. (vi) L-kynurenine (Sigma) was reconstituted at a final concentration of 0.50 mM in H_2_O. (vii) NAC (Sigma) was made by dissolving in H_2_O for a final concentration of 5 µM. (viii) For 200 µM of melatonin (*N*-Acetyl-5-methoxytryptamine, Sigma), 10 mg was dissolved in 430 µl ethanol, and 20 µl was used for 1 ml culture. (ix) From 10 mM of tBHP (Sigma, 1 mg/1.11 ml H_2_O), 0.1 µl (1 µM) was used for 1 ml culture. (x) For 500 µM of teriflunomide (Tocris), 50 µl of the stock solution in DMSO was used in 1 ml cell culture.

### Cell culture and infection

The human intestinal epithelial cell line Caco2, and SW480 were cultured with Dulbecco’s minimum essential media (DMEM) with 10% bovine serum. Mouse macrophages (RAW-264.7) were cultured in RPMI, and bone-marrow dendritic cells from mouse bone marrow were differentiated in RPMI with 10% serum in the presence of recombinant GM-CSF (10 ng/ml) and IL-4 (20 ng/ml)^10^.

*T. gondii* RH strain was grown in human foreskin fibroblasts cells^53,54^ and purified by differential centrifugation (3000×*g*, 10 min). Parasites were resuspended in phosphate-buffered saline (PBS), counted in a hemocytometer under microscope, and used for infection in different cells at MOI of 2. Parasite growth was measured by immunoblot using *T. gondii* specific antibody (SAG1) as well as qPCR using specific primers^10^. For confocal microscopy, mCherry was tagged endogenously with hexokinase of *T. gondii* and the transformed parasites were selected and grown in the presence of pyrimethamine.

### Plasmids and transfection study

The FLAG-tagged human wild-type (Wt)-β-catenin, phospho-mutant S552A-β-catenin, constitutively active-S552D-β-catenin, kinase-inactive phosphorylation-deficient AKT1/2 (K179A/T308A/S473A, or HA-AKT1/2-AAA dominant-negative) and constitutively active AKT1/2 (HA-PKB-308D/473D or AKT1/2-DD) constructs were kind gifts from Zhimin Lu, University of Texas M. D. Anderson Cancer Center, USA^3^. Human pEGFP-β-catenin, and FLAG-hTCF4 plasmids were kindly provided by Vitezslav Bryja, Masaryk University, Czech Republic, and human FLAG-tagged IDO1 in pEF-BOS plasmid was kind gift from Ciriana Orabona, Italy. Caco2 cells were transfected with lipofectamine 3000 (Invitrogen) following manufacturer’s instructions. For primary DCs, plasmid was transfected using lipofectamine LTX and Plus reagent (Invitrogen). Unless otherwise stated, 0.8 μg plasmid was used for transfection per 5 × 10^5^ cells in each 4 cm^2^ well of the plate. After 8 h post transfection, cells were infected with *T. gondii* at a MOI of 2.

### Reporter assay

To prepare the minimal promoter (MinP) construct containing the TCF4-binding site of the IRF3 promoter (CTTTGGG/CTTTGCG), a nonspecific 2.7 kb PCR product was cloned into pGL3 vector between NheI and HindIII sites to generate TCF4-MinP-PGL3. The MinP sequence (32 bp) was then inserted 68 bp upstream of the luciferase gene by inverse PCR. Clones were confirmed by the ~2 kb fragment generated upon restriction with EcoRI and BamHI, since the EcoRI site is present in the MinP sequence and BamHI site is in the vector backbone. The confirmed vector was used for insertion of the 49 bp TCF4 sequence (TTTGCGCTTTGGGCTTTGGGCTTTGGGCTTTGGGCTTTGGGCTTTGGG) at 2.5 kb upstream of MinP sequence by inverse PCR. The final clone was confirmed by restriction digestions with the respective enzymes, followed by sequencing. M50 Super 8x Top-Flash (Top-Flash, addgene-12456) where seven TCF/LEF binding sites are present upstream of firefly luciferase reporter, and M51 Super 8x Fop-Flash (Top-Flash mutant, addgene-12457), where mutated TCF/LEF binding sites are present upstream of luciferase reporter^55^, were used for luciferase reporter assay. The firefly luciferase reporter construct under the control of IFN-β promoter (p125-Luc) was kindly provided by Ganes Sen, Cleveland Clinic Foundation, USA and human IDO1 reporter plasmid was generous gift from George C. Prendergast, Lankenau Institute for Medical Research, USA. The p125-luc and IDO1 promoters were activated by IFN-β (500 units/ml) and IFN-γ (1000 units/ml), respectively. IDO promoter activity was checked in the presence of phospho-mimic constitutively active IRF3 (Ser396-398-402-405-Asp, Thr-404-Asp) and phospho-mutant IRF3 (Ser-396-398-Ala)^56,57^. Caco2 cells were seeded in 2 cm^2^ well of a plate, and were transfected with 50 ng of the luciferase reporter plasmid together with a total of 200 ng of various expression plasmids or empty control plasmids and pCMV-Renilla-Luc vectors (50 ng). *Renilla* luciferase plasmid was used as an internal control in all luciferase assays. After 8 h of transfection, cells were infected with *T. gondii* for 12 h and luciferase activity in the total cell lysate was measured by the Dual-Luciferase Reporter Assay kit (Promega). All experiments were performed in triplicates.

### Human β-catenin and IDO gene targeting

β-catenin CRISPR/Cas9 knock-out plasmid (gene locus: human-CTNNB1 mapping to 3p22.1) was designed to disrupt gene expression by causing a double-strand break (DSB) in a 5′ constitutive exon within the CTNNB1 (human) gene. The β-catenin CRISPR/Cas9 KO plasmid kit (Santacruz; sc-400038) consists of a pool of 3 plasmids, each encoding the Cas9 nuclease and a target-specific 20 nt guide RNA (gRNA) designed for maximum knockout efficiency. The β-catenin HDR plasmid (h) (Santacruz, sc-400038-HDR) was used for selection of cells containing a DSB induced by β-catenin CRISPR/Cas9 KO Plasmid (h). The IDO CRISPR/Cas9 KO plasmid was designed in a similar manner (Santacruz, sc-400495, & sc-40095HDR). At 12 h post-transfection, cells were positively selected in puromycin (2 µg/ml) for 72 h, and the culture was maintained in the presence of puromycin. The deletion of gene was confirmed by both immunoblotting and PCR.

### Immunoprecipitation (IP) and immunoblot

For immunoprecipitation, after transfection in 9 cm^2^ well of the plate, and followed by infection, cells were lysed in lysis buffer containing 20 mM HEPES (pH 7.4), 50 mM NaCl, 1.5 mM MgCl_2_, 2 mM DTT, 2 mM EGTA, 10 mM NaF, 12.5 mM β-glycerophosphate, 1 mM Na_3_VO_4_, 5 mM Na_4_P_2_O_7_, 0.2% (v/v) Triton X-100, and protease inhibitors (Himedia). Cell lysates, containing 400 µg of total protein were precleared with mouse immunoglobulin G (IgG) agarose (Sigma) in IP buffer for 1 h, and then incubated overnight with IP-specific antibodies, followed by protein A/G beads for 1 h with rotation. After incubation, the beads were washed with 1 × PBS, and protein complexes were eluted by adding 40 µl of 2 × sample buffer to each IP reaction and heating at 50 °C for 15 min. For immunoblot, cells were lysed in the 1.5 × Laemmli sample buffer containing protease inhibitor cocktails (Himedia) and for phospho-antibody, phosphatase inhibitors cocktail (Cell Signalling Technology) were additionally used. After sonication, samples were heated at 95 °C and equal amount of proteins were analysed on denaturing SDS-polyacrylamide gels after estimation of protein concentration. The proteins were transferred to PVDF blotting membrane (Amersham Hybond) and probed with specific primary antibody, followed by horseradish peroxidase (HRP) conjugated secondary antibody. Bands were visualized by chemiluminescence-based detection system (Amersham) using SuperSignal West Femto/Pico Plus substrate (Thermo-Fischer Scientific). The intensity of protein bands was quantified using ImageJ software, NIH, USA^58^, and β-actin was used as control for normalization. Statistical bar diagram of each immunoblot was generated from three or more repeat experiments after normalizing with the β-actin band. The following antibodies were used as and where needed. Phospho-β-catenin (Cell Signalling, #9566), β-catenin (Cell Signalling, #9562), *T. gondii* SAG1/p30 (Abcam TP3 monoclonal, #ab8313), FLAG (Sigma, #3165), Phospho-AKT-Ser-473 (Cell Signalling, #9271), AKT-pan (Thermo Fischer, #44609), α**-**tubulin (Abcam, #7291), histone H3 (Abcam, #1791), phospho-IRF3 (Cell Signaling, #4947), Phospho-STING (Cell Signalling, #19781), Phospho-TICAM2 (Fabgennix, #PTRAM-140AP), IDO1 (Biolegend, #695001), TDO (Biocompare, #134311), HA (Santacruz, #805), ubiquitin (Cell Signalling, #3933), β-actin (Abcam, #8227), mouse secondary-HRP (Santacruz, #2031), and rabbit secondary-HRP (Santacruz, #2030).

### Ubiquitination assay

To analyse the ubiquitination of IDO1, Caco2 cells were transfected with FLAG-tagged wild-type-IDO1 (FLAG-Wt-IDO1), or FLAG-IDO1-Y-115-F mutant, or FLAG-IDO1-Y-253-F mutant, or FLAG-IDO1-Y-115-115-F double mutant plasmid. After 12 h of transfection, the cells were pre-treated with proteasome inhibitor (MG132, 10 µM) for 4 h, and then infected with parasites for 6 h in the presence of MG132. The cell lysates were immunoprecipitated with FLAG antibody and the immunoprecipitates were analysed by immunoblot, using anti-ubiquitin. The above mentioned 4 plasmids were kindly gifted by Dr. Paolo Puccetti, University of Perugia, Italy.

### RNA extraction and qPCR

Total RNA from Caco2 cells was isolated using TRI Reagent (Sigma), and concentration was measured. The purity and integrity of RNA was checked after DNase treatment. cDNA was made from 1 μg of RNA using the revert aid first strand cDNA synthesis kit (Thermo Fisher Scientific). For qPCR, the PCR mixture (total volume 6 μl) was prepared containing 3 μl of SYBR AmpliTaq Gold DNA Polymerase (ABI), 1 μl of cDNA, forward and reverse primers (0.2 μl each) and DEPC water (1.6 μl). The primers used for qPCR are listed below. For *β-catenin*- Fwd: AGAACCCCTTGGATATCGCC, Rev: TGGCCACCCATCTCATGTTC, *IDO1*- Fwd: GCCTGTGTGAAAGCTCTGGTC, Rev: CCTCCAGTTCCTTTGGCTTCC, *TDO*- Fwd: CTTAGTAAAGGTGAAAGACGG, Rev: GTCCATAAGAGAAGTCAGCA, *IRF3*- Fwd: TCTGCCCTCAACCGCAAAGAAG, Rev: TACTGCCTCCACCATTGGTGTC, and *β-actin*- Fwd: AGTCATTCCAAATATGAGATGCGTT, Rev: GCTATCACCTCCCCTGTGTG. The comparative Ct (ΔΔCt) method was used to calculate the expression of target genes using Real-Time PCR (ABI ViiA7; Applied Biosystems, USA), and β-actin was used as the internal calibrator. The fold change in expression was used as a relative measure of gene expression. For determining the growth of *T. gondii*, qPCR was performed using primer pair, against the ITS-1 region conserved in all *T. gondii* strains: Fwd: AATATTGGAAGCCAGTGCAGG, Rev: CAATCTTTCACTCTCTCTCAA^59^. Primers for *T. gondii* ITS-1 gene did not generate a product when the genomic DNA was used as template derived from uninfected cells.

### Microscopic study

For both confocal and fluorescence microscopy, cells (2 × 10^5^ /well) were plated onto cover glasses in 9 cm^2^ well of the plates, grown overnight (to ~5 × 10^5^ cells). For fluorescence microscopy, cells were transfected with GFP-tagged wild-type (Wt) or phospho-mutant, or constitutive-active β-catenin plasmids. After 12 h of transfection, cells were then infected with *T. gondii*. Different types of cells were similarly infected with the same number of parasites for confocal microscopy. At indicated times post-infection, cells were fixed in ice-cold methanol for 5 min and permeabilized with PBS containing 0.1% Triton X-100. Fixed cells were blocked in PBS containing 1% BSA for 1 h and the nucleus was stained with DAPI. Mounting was done using fluoroshield (Sigma), and cells viewed under a fluorescence microscope (×100; Axio-Imager, Z2; Carl Zeiss Iberia). For confocal microscopy, mCherry expressing *T. gondii* was used for the infection. Methanol fixed cells were blocked in PBS containing 1% BSA for 1 h. Nuclei were stained with DAPI. Cells were visualized at a ×60 magnification in a Nikon Eclipse Ti2 confocal microscope.

### Preparation of nuclear and cytoplasmic fractions

Cells were transfected with FLAG-tagged wild-type (FLAG-Wt-β-catenin), or phospho-mutant (FLAG-β-catenin-S552A) or constitutive active (FLAG-β-catenin-S552D) plasmid and after 12 h of transfection, infected with parasites. Cells were collected at different times post-infection and nuclear and cytosolic fractions were purified using NE-PER-nuclear and cytoplasmic extraction reagents kit (Thermo-Fischer Scientific).

### Enzymatic activity of IDO1

*T. gondii*-infected Caco2 cells (1 × 10^6^ cells) treated with or without IFN-γ in the presence of L-tryptophan were harvested at different time points after infection. Culture supernatants were collected and adherent cells were isolated by trypsinization. Cells were washed three times in PBS. Both cells pellet and culture supernatant were used for IDO1 activity assay. This assay could measure both IDO1 and IDO2, but as the IDO2 expression was undetectable in the course of *T. gondii* infection, so we assumed that production of kynurenine from tryptophan was due to functional IDO1 activity. Cell extracts (100 µl) were mixed with an equal amount of reaction buffer (100 mM potassium phosphate buffer pH 6.5, 40 mM ascorbate, 20 mM methylene blue, 200 mg/ml catalase, 800 µM L-tryptophan). The mixture was incubated for 30 min at 37 °C. Thereafter, the reaction was stopped by adding 40 µl of 30% (w/v) trichloroacetic acid and further incubated at 50 °C for 30 min. After centrifugation (6000×*g*, 10 min), 100 µl of supernatant was mixed with 100 µl Ehrlich reagent (100 mg, p-dimethylamino-benzaldehyde/5 ml, glacial acetic acid; Sigma) in a 0.3 cm^2^ well of microtiter plate. Samples were run against a standard curve of defined kynurenine concentrations (0–2.5 mM; Sigma). Absorbance at 492 nm was read with a microplate reader within 10 min. The change in kynurenine concentration was obtained by subtracting control levels (uninfected culture supernatant) from the sample value using the standard curve.

### Quantification of melatonin

Caco2 cells (1 × 10^6^ cells/ml) were infected with *T. gondii* for different time periods at 2 MOI, and infected cells were treated with either kynurenine or its analogue (teriflunomide) in separate sets. Cell supernatants were collected at indicated time points, and melatonin was quantified using commercial ELISA kit (Cusabio)^60^.

### Measurement of ROS

In addition to their critical role in ATP synthesis, mitochondria are also major source of ROS in cells. It has been suggested that 2% of the oxygen consumed by mitochondria is converted to superoxide. In turn, superoxide is converted by manganese superoxide dismutase to H_2_O_2_. We used Amplex red hydrogen peroxide/peroxidase kit (Thermo Fischer Scientific) to detect H_2_O_2_ in 0.3 cm^2^ well of the plate with 100 µl of total volume^61^. The method uses 10-acetyl-3,7-dihydroxyphenoxazine (10 µM) in combination with HRP. H_2_O_2_ released from cell, reacts with 10-acetyl-3,7-dihydroxyphenoxazine in the presence of peroxidase and generates resofurin (red-fluorescent oxidation product), which is detected at excitation and emission of 571 nm and 585 nm, respectively. The concentration of H_2_O_2_ was determined with the help of a standard curve prepared during experiment.

### Apoptosis study

Nuclear DNA fragmentation is a hallmark of apoptosis. Genomic DNA was isolated from *T. gondii* infected Caco2 cells that were treated with kynurenine or its analogue, teriflunomide; untreated-uninfected cells served as control. The extracted DNA was electrophoretically separated on 1.8% agarose gel in the presence of molecular size markers (100 bp) and visualized after staining with 0.5% ethidium bromide (Sigma).

### Statistical analysis

Changes between treatment groups of cells or between sets of experiments were analysed by one-way ANOVA and by Students *t*-test. All numerical data were collected from at least three separate experiments. Results were expressed as mean ± SE (standard error bars in graphs). *p* values < 0.05; <0.01; and <0.001 were considered statistically significant.

## Supplementary information

Supplementary Figure S1. % intracellular parasite load and % formation of PVs

Supplementary Figure S2. Parasite growth

Supplementary Figure 3. Working model
