# Supplementary figures and images for: Tryptophan-kynurenine pathway attenuates β-catenin-dependent pro-parasitic role of STING-TICAM2-IRF3-IDO1 signalosome in Toxoplasma gondii infection
Source: Cell Death Dis. 2019 Feb 15;10(3):161. doi: 10.1038/s41419-019-1420-9 (PMC6377608; doi:10.1038/s41419-019-1420-9)

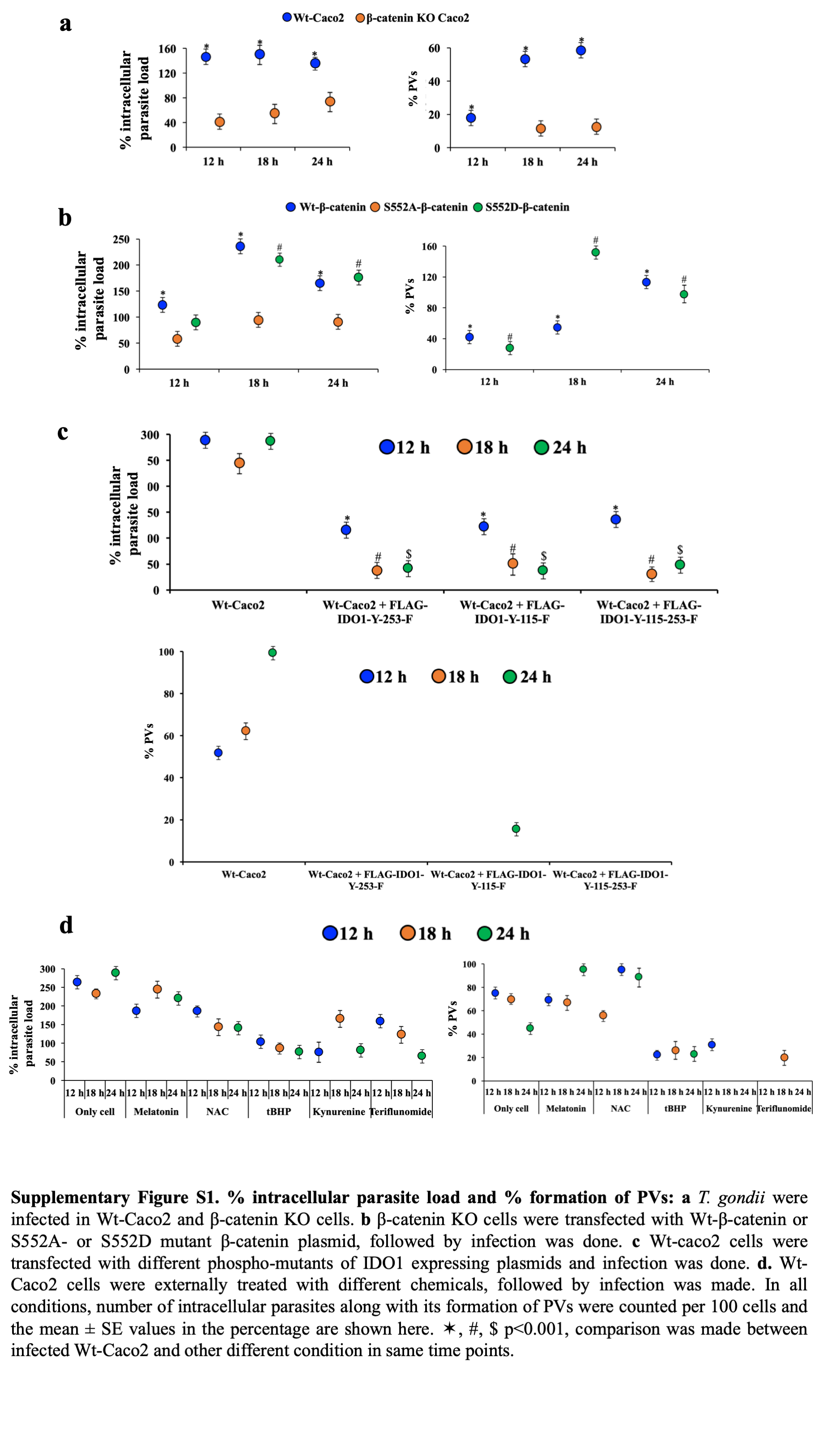

Supplement: Supplementary file 1 — Supplementary Figure S1. % intracellular parasite load and % formation of PVs [file 41419_2019_1420_MOESM1_ESM.tif]

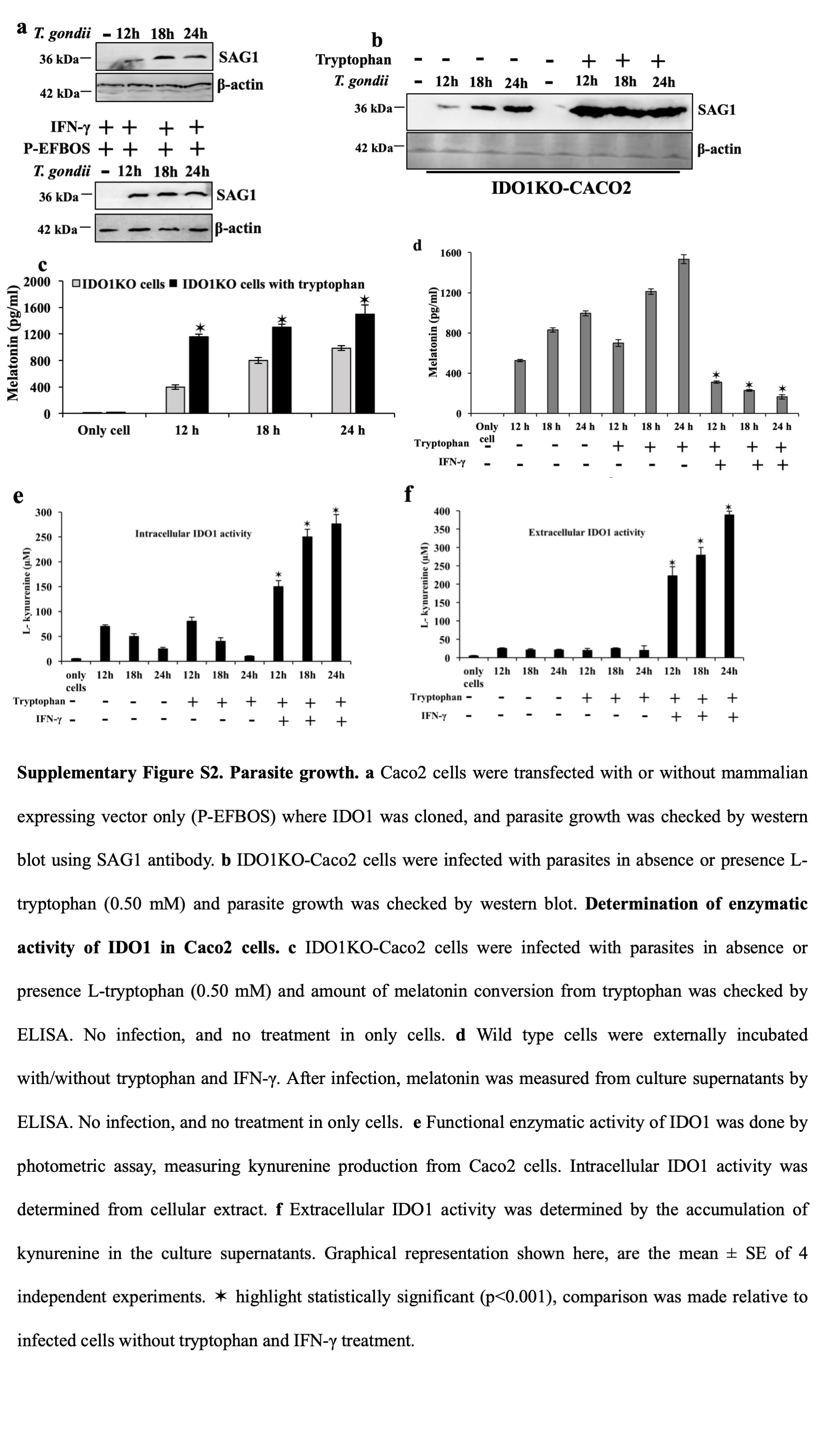

Supplement: Supplementary file 2 — Supplementary Figure S2. Parasite growth [file 41419_2019_1420_MOESM2_ESM.tif]

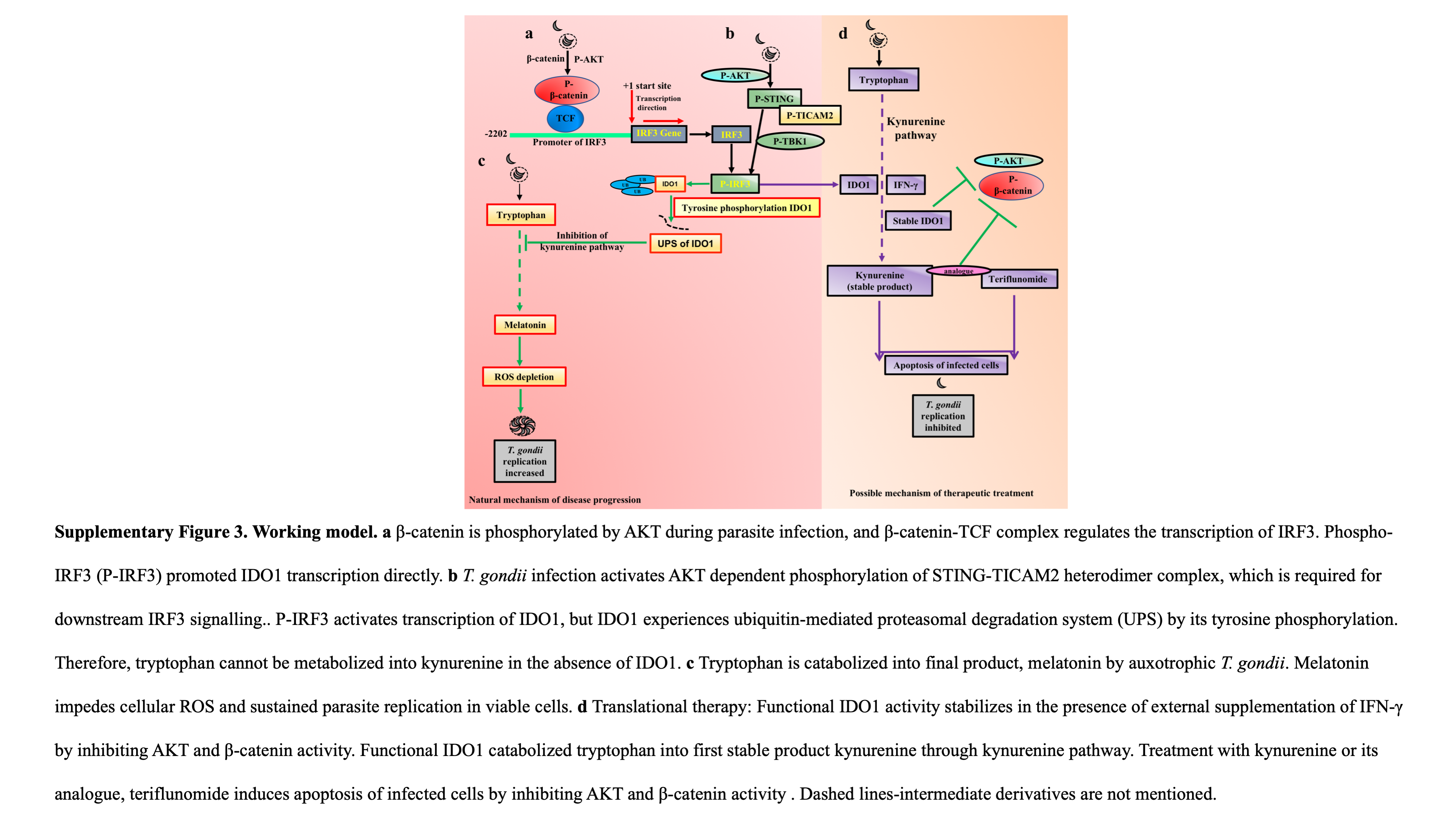

Supplement: Supplementary file 3 — Supplementary Figure 3. Working model [file 41419_2019_1420_MOESM3_ESM.tif]
